# An Optical Wavefront Sensor Based on a Double Layer Microlens Array

**DOI:** 10.3390/s111110293

**Published:** 2011-10-31

**Authors:** Vinna Lin, Hsiang-Chun Wei, Hsin-Ta Hsieh, Guo-Dung John Su

**Affiliations:** Graduate Institute of Photonics and Optoelectronics, National Taiwan University, No. 1, Roosevelt Road, Section 4, Taipei, Taiwan; E-Mails: r98941025@ntu.edu.tw (V.L.); d96941017@ntu.edu.tw (H.-C.W.); htjhsieh@gmail.com (H.-T.H.)

**Keywords:** microlens array, long focal length, Shack-Hartmann wavefront sensor

## Abstract

In order to determine light aberrations, Shack-Hartmann optical wavefront sensors make use of microlens arrays (MLA) to divide the incident light into small parts and focus them onto image planes. In this paper, we present the design and fabrication of long focal length MLA with various shapes and arrangements based on a double layer structure for optical wavefront sensing applications. A longer focal length MLA could provide high sensitivity in determining the average slope across each microlens under a given wavefront, and spatial resolution of a wavefront sensor is increased by numbers of microlenses across a detector. In order to extend focal length, we used polydimethysiloxane (PDMS) above MLA on a glass substrate. Because of small refractive index difference between PDMS and MLA interface (UV-resin), the incident light is less refracted and focused in further distance. Other specific focal lengths could also be realized by modifying the refractive index difference without changing the MLA size. Thus, the wavefront sensor could be improved with better sensitivity and higher spatial resolution.

## 1. Introduction

An optical wavefront sensor is an important device in determining wavefront aberration if fields ranging from the astronomy to any optical testing application. There are several types of wavefront sensors including the Michelson interferometer, Shearing interferometer, Fizeau interferometer, and even the Foucault knife-edge test. Among these interferometric methods, the Shack-Hartmann wavefront sensor (SHWS) is the most popular sensor because of its simplicity and elegance for measuring the shape of a wavefront. It has been applied to adaptive optics for high-energy lasers and astronomy for many years [1]. The main goal is to improve image quality taken by ground-based telescopes which might be distorted by atmospheric turbulence. However, as the technology has been developed, the technique has been implemented in many other fields. Over time, applications in quality laser beam measurement, optics testing, and optical system calibration and alignment have been discussed. Furthermore, this technology quickly led to the evolution of more sophisticated sensors focused around ophthalmic applications [2], CCD cameras, and micro-optics. The applications of SHWS have become widespread throughout the World, with hundreds of millions of astronomical images benefiting from the process to millions of corrective surgeries that will be performed in upcoming years to enhance vision. It is amazing for a technology to have such a dramatic impact and evolution from a single field to multiple fields as the SHWS has.

The most critical element of an SHWS is its microlens array (MLA). The incident light is divided into a number of small samples by the lenslet arrays, which then are focused onto a detector array. These focal spots of light are the key principle in the measurement of wavefront aberration. The wavefront slopes are computed by the displacement of the measured focal spots from their reference spot positions. In this paper, we first propose a design and method for extending the focal length of MLA effectively based on double layer structure; the evaluation of the MLA is presented accordingly. Furthermore, we integrated the MLA with an image sensor to build a SHWS. Not only the development of the SHWS is discussed but also the experimental results of the SHWS performance is explained by comparing the measured wavefront with the commercial one. The experimental results of the system are discussed and compared between the long focal length, the shorter focal length and the commercial SHWS.

## 2. Design and Simulation

In a thermal reflow process, microlenses are made by heating photoresist cylinders. The focal length (*f*) of MLA could be described as in Equation (1) [3]:

(1) $$\begin{array}{l} f=\frac{r_{c}}{n-1}\\ r_{c}=\frac{h^{2}+r^{2}}{2h} \end{array}$$

where *n* is the refractive index of MLA material, *r_c_* is radius of curvature, which is related to the height, *h* and radius, *r* of melted MLA [4], the lens profile is assumed to be a spherical shape here. However, only some profiles are possible. If the ratio of height to diameter is too low, photoresist cylinders only reflow at the edge of itself. In other words, photoresist cylinders would not become microlens arrays. The ratio of lens height to diameter should not be too small. Although increasing diameter does increase maximum focal length, the thickness of the photoresist also needs to be increased. A thick photoresist is hard coat uniformly and multi-coatings increase the complexity of the process, so there is a trade-off between a long focal length and photoresist thickness. Therefore, we propose a new design to extend the focal length based on the calculations that follow.

**Figure 1.**
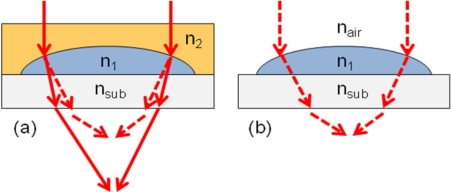
**(a)** Light propagation in a microlens by **(a)** our new method (solid lines) and **(b)** traditional thermal method (dashed lines).

Figure 1 shows the schematic drawing of our new design. An additional cover layer is applied on the top of MLA. The light is less refracted at the MLA surface as shown in Figure 1(a) and this results in the extension of the focal length. The new MLA design consists of a plano-concave lens and a following plano-convex lens. The total focal length is described by Equation (2) [5]:

(2) $$\frac{1}{f}=\frac{1}{f_{1}}+\frac{1}{f_{2}}-\frac{d}{f_{1}\cdot f_{2}}$$

Parameter d is the separate distance, which is zero in our case. Substituting Equation (1) into *f*_1_ and *f*_2_, we can get the effective focal length as in Equation (3). Notice the focal length of cover layer or plano-concave lens is negative.

(3) $$f_{\mathrm{eff}}={\left({\left(\frac{r_{c}}{n_{1}-1}\right)}^{-1}+{\left(\frac{-r_{c}}{n_{2}-1}\right)}^{-1}\right)}^{-1}=\frac{r_{c}}{n_{1}-n_{2}}$$

**Figure 2.**
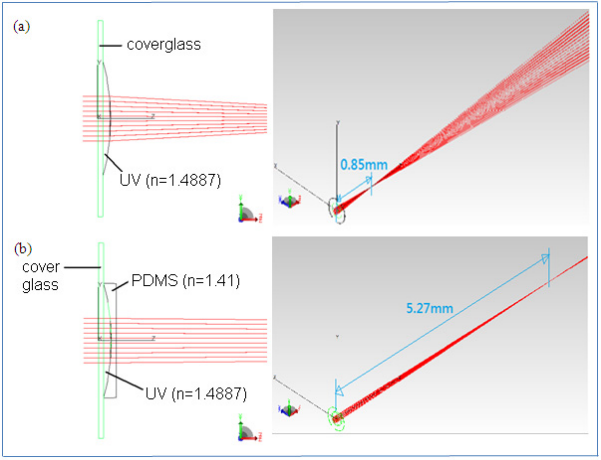
Ray tracing simulation by Tracepro^®^ of 235 μm diameter MLA **(a)** without cover layer and **(b)** with a PDMS cover layer on it.

Comparing focal length in Equations (1) and (3), the denominator is reduced from (*n*_1_ − 1) to (*n*_1_ − *n*_2_). The extending factor of focal length is determined by the ratio of (*n*_1_ − 1) over (*n*_1_ − *n*_2_). Finally, we show a TracePro^®^ ray tracing plot of focal length extension over a spherical case in Figure 2. We set UV-resin (*n* = 1.4887) as our lens material and PDMS (*n* = 1.41) as our cover layer material. The focal length is indeed increased after a cover layer was added. Based on the simulation result, the focal length of 235 μm diameter MLA is expected to be extended from 0.85 mm to 5.27 mm, which is six times longer than original one. This agrees with our previous design idea.

## 3. Fabrication Processes and Discussion of Results

**Figure 3.**
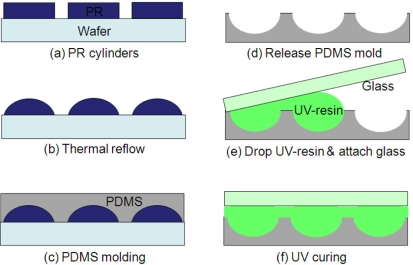
Fabrication steps of long-focal length MLA.

The long-focal-length MLA was made of polymer materials. We fabricated an opaque photoresist MLA first and transferred it into a transparency MLA with a cover layer. The fabrication steps are illustrated in Figure 3. In photoresist MLA fabrication, the silicon wafer was cleaned by piranha (H_2_O_2_ + H_2_SO_4_) solution and dehydrated by baking at 120 °C for 10 minutes. Then, we spun a layer of photoresist (AZ-P4620) at 1,000 rpm for 60 seconds on the silicon substrate, which resulted in a 13 μm thick photoresist. The photoresist cylinders were patterned after photolithography as seen in Figure 3(a). After heating at 160 °C for 10 hours, photoresist cylinders formed MLA with a spherical profile as seen in Figure 3(b). The next step was the transfer process, which replicated photoresist MLA to a new MLA made of UV-resin. As shown in Figure 3(c), we poured liquid polydimethysiloxane (PDMS, 184 from Dow Corning) on photoresist MLA followed by a low-speed spin of 100 rpm to reduce the thickness. After spin coating, the PDMS replicated the surface profile of MLA (e.g., PDMS was conjugated to the MLA surface). The PDMS coated wafer was relaxed for 10 minutes to reduce roughness. Once the top of liquid PDMS became flat after standing for 10 minutes, it was heated and cured at 65 °C for four hours. After cooling to room temperature, the PDMS mold was removed from the photoresist MLA. Because of low surface free energy and elasticity of PDMS [6], it could be released from MLA easily without anti-stiction issue. Figure 3(d) shows a negative or concave master mold after releasing. Then, a drop of UV-resin (GA-126) was put on a PDMS mold and the mold was attached to a cover glass as shown in Figure 3(e). The UV-resin liquid went outward and filled the whole MLA area. We did this step carefully on a flat optical table without any air bubbles between the PDMS mold and a cover glass. This makes sure only a thin UV-resin layer was left. After UV curing, long-focal-length microlens arrays were fabricated on a cover glass as shown in Figure 3(f).

**Figure 4.**
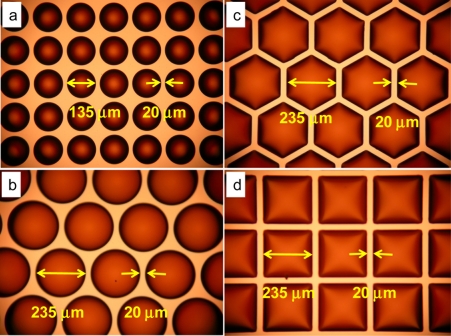
The picture of the photoresist of: **(a)** circular lens square grid MLA, **(b)** circular lens hexagonal grid MLA, **(c)** hexagonal lens MLA, and **(d)** square lens MLA after thermal reflow.

**Figure 5.**
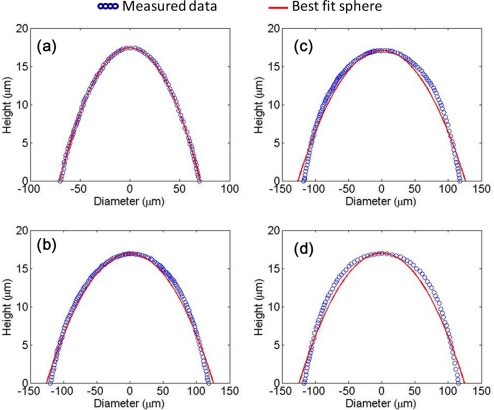
Lens profile of: **(a)** circular lens quadratic grid (d = 135 μm), **(b)** circular lens hexagonal grid (d = 235 μm), **(c)** hexagonal lens (d = 235 μm), and **(d**) square lens (d = 235 μm) by probe-type surface analyzer. The circles show measured points and solid lines are best fit spheres.

For comparison, different sizes with different structures and arrangements of MLAs were fabricated on the same wafer which consisted of: circular lens quadratic grid (d = 135 μm), circular lens hexagonal grid (d = 235 μm), hexagonal lens (d = 235 μm), and square lens (d = 235 μm). Figure 4 shows the MLAs after the thermal reflow process. The microlenses in Figure 4 were fully reflowed and the height of the microlenses is about 17 μm. Figure 5 shows the profile of Figure 3 as determined by a probe-type surface analyzer (KOZAKA, ET-4000A).

We assumed that the MLA is a sphere in the calculation. However, the profile of reflowed microlens is aspherical as the measured data in Figure 5 show. The solid line represents the best fit sphere. We approximated the value of MLA surface coordinates to the sag equation without high order term as in Equation (4) [7]:

(4) $$z(r)=\frac{{\mathrm{cr}}^{2}}{1+\sqrt{1-(1+k)c^{2}r^{2}}}$$

where *c* is curvature at the vertex, as usual *c* = *1/r_c_*, *k* is conic constant defined the type of surface curvature, *r* is the radial coordinate measured perpendicular from the axis and *z* is function of the distance from lens center *r*. After curve fitting on the measured data, the conic constant *k* is about −0.24 and resulted in a 5.24 mm focal length in the calculation. The focal length was indeed increased after a cover layer was added. No significant change on the focal length was seen between the spherical and aspherical case. Besides, for parallel incident rays, less reflection is observed in the double layer case due to the lower refractive index difference at the boundary.

**Figure 6.**
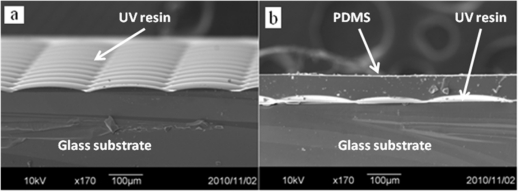
SEM image of the UV-resin MLA **(a)** without and **(b)** with the second layer of PDMS.

**Figure 7.**
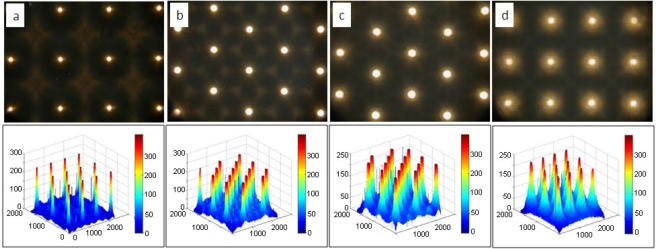
The focused spot images (above) and their intensity distribution (below) of **(a)** circular lens quadratic grid MLA, **(b)** circular lens hexagonal grid MLA, **(c)** hexagonal lens MLA, and **(d)** square lens MLA.

Figure 6 is the scanning electron microscope (SEM) image of the UV-resin MLA without and with the covering layer of PDMS. There are three layers shown in Figure 6(b) from bottom to top: a glass substrate, UV-resin, and PDMS film as the flip structure in Figure 3(f). In addition, the UV-resin layer could also be replaced by other polymers such as SU-8 photoresist with different refractive index. The resulting focal length was then determined by the refractive index difference. In this fabrication, the refractive index n_1_ of UV resin (microlens layer) was 1.4887 and the refractive index n_2_ of PDMS (covering layer) was 1.41. We measured the focal spot experimentally by moving the optical microscope up and down. The distance between the light-focusing point and vertex of the microlens was approximately 5.25 mm for a 235 μm diameter microlens and 2 mm for a 135 μm diameter microlens. Because of its high NA objective lens, the depth of focus (DOF) of a microscope is very small (∼0.7 μm). The accuracy of the image plane position is around ±0.7 μm. The focused spot patterns and their intensity distribution are shown in Figure 7. The spot was measured under a microscope (ECLIPSE 50i with 50 × objective, Nikon) with bottom emitting light source. The spot profiles were symmetric, which means that each UV lens in the MLAs has good quality. In other words, the fabricated microlenses are symmetric. The profile of our microlenses is close to a sphere near the vertex and the side part of the microlenses is differs slightly from a spherical curve.

The fill factor of MLAs is related to the arrangement and gap of each lens as shown in Table 1. It shows that a square lens and a hexagonal lens have the same fill factor and a circular lens with a square arrangement has the lowest fill factor. The gap between each lens in our experiment was 20 μm. The fill factor could be increased by decreasing the gap distance of each lens.

Finally, we show the comparison of methods which could increase the focal length by modifying different variables in Table 2. Unlike other methods that increase the focal length slightly (∼1X), the method in this paper produces a significant increment (∼6X) in the focal length. Although two material layers are required, the second PDMS coating process is easy and could be done at room temperature. The room temperature curable properties of PDMS prevent any temperature variation during the processes, which minimizes the residual stress due to coefficient of thermal expansion (CTE) mismatch. Besides, the cured PDMS has good chemical stability. It also provides a passivation layer, which protects the inner microlens material from water absorption or chemical attack. In addition, the flat MLA surface is easier to clean than an uneven surface (e.g., the original single layer MLA without a passivation layer).

## 4. Wavefront Sensor Computing Algorithm and Measurement Results

The Shack-Hartmann wavefront sensor utilizes a microlens array to divide and reconstruct the incident wavefront. The incident light is divided into a number of beamlets by the two-dimensional microlens array [8]. The spot displacement which is related to the wavefront slope could be calculated by comparing the reference focal spots and the measured wavefront focal spots provided by each lenslet. Each local wavefront slope corresponds to a wavefront distortion. The relationship between incoming wavefront slope *θ*, spot displacement Δ*d*, and focal length of the lenslet *f* can be described by Equation (5) [7]:

(5) $$\theta =\frac{\Delta d}{f}$$

There are various methods for reconstructing the wavefront from the slope measurements. We chose modal reconstruction in our calculation. The wavefront is described in terms of functions that have analytic derivatives in the modal reconstruction method. The measured slope data was then fit to the derivative of these functions, allowing a direct determination of the wavefront from the fitting coefficients. Therefore, it is desirable to expand the wavefront aberration in terms of a complete set of basis functions that are orthogonal, such as Zernike polynomials *Z_k_*(*x*,*y*) [9]:

(6) $$w\left(x,y\right)=\sum_{k=1}^{+\infty }\omega_{k}\;Z_{k}\left(x,y\right)$$

where *ω_k_* represents the Zernike coefficient. So we can write the local wavefront slopes as in Equation (7):

(7) $${\left(\begin{array}{c} \partial w/\partial x\\ \partial w/\partial y \end{array}\right)}_{m}=\left(\begin{array}{c} \sum_{k=1}^{+\infty }\omega_{k}\frac{\partial Z_{k}}{\partial x}\\ \sum_{k=1}^{+\infty }\omega_{k}\frac{\partial Z_{k}}{\partial y} \end{array}\right)$$

We can rewrite Equations (5) and (7) as following matrix:

(8) $$\left[\begin{array}{c} \theta_{x}(1)\\ \cdots \\ \theta_{x}(p)\\ \theta_{y}(1)\\ \cdots \\ \theta_{y}(p) \end{array}\right]=\left[\begin{array}{ccc} g_{1}(1) & \cdots & g_{k}(1)\\ \cdots & \cdots & \cdots \\ g_{1}(p) & \cdots & g_{k}(p)\\ h_{1}(1) & \cdots & h_{k}(1)\\ \cdots & \cdots & \cdots \\ h_{1}(p) & \cdots & h_{k}(p) \end{array}\right]\;\left[\begin{array}{c} \omega_{1}\\ \cdots \\ \omega_{k} \end{array}\right]$$

where *p* is the number of centroid positions in this calculation. Therefore, we can calculate the unknown Zernike coefficient, *ω_k_* by the inverse and the transpose of above matrix. By the matrix operation, we could get each of the corresponding Zernike polynomial wavefront aberration coefficients and take them into Equation (6). The working principle of the Shack-Hartmann wavefront sensor included the recognition of the aperture size/lens focal length, the detection of the image spots for reference beam and distorted beam, the calculation of the spot center and the displacement, the calculation of the Zernike coefficients from the wavefront, and the reconstruction of wavefront.

**Figure 8.**
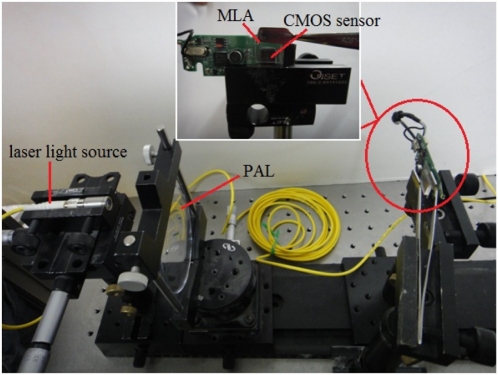
The experiment setup for measuring a wavefront aberration of testing lens (PAL) by using our fabricated MLA and SHWS.

In order to measure the wavefront aberration, we integrated our fabricated MLA with an image sensor to construct a Shack-Hartmann wavefront sensor. We show the whole experiment setup in Figure 8. The laser light source from optical fiber is collimated by an aspherical lens screwed in front of the fiber connector. The collimated laser produces a reference wavefront which passes through our microlens array and results in separated focal spots on the detector. In this set up, we chose our fabricated hexagonal shaped MLA to focus the light on sensor because it not only has a higher fill factor, but also higher reconstruction precision.

To evaluate our SHWS performance, testing samples such as PAL (progressive addition lens) and PCX (plano-convex lens) was measured by our wavefront sensor. The test samples could produce wavefront aberrations when they are placed between the laser light source and the SHWS.

**Figure 9.**
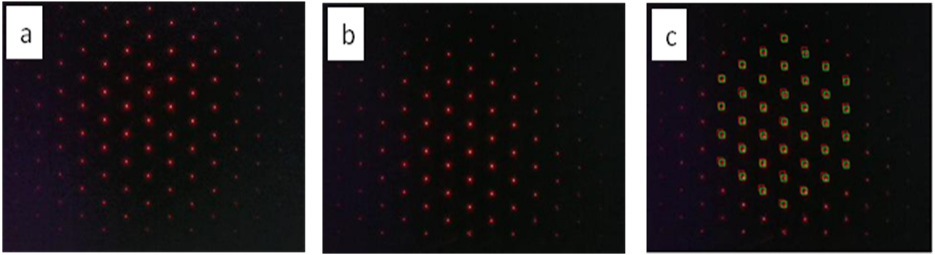
**(a)** Focal spots image of the reference wavefront, **(b)** focal spots image of the aberrated wavefront, and **(c)** detection picture of image spots for reference beam and distorted beams.

For the measurement, we first took pictures of focal spots without aberration from the test samples as the reference points, as shown in Figure 9(a), and then a second picture of focal spots with aberration by putting the test lens between the laser light source and our SHWS, as shown in Figure 9(b). In order to calculate the centroid spots precisely, a hexagon mask is defined to cover only one point in every calculation. We calculated 37 wavefront slopes of focal spots taken by the CMOS (complementary metal–oxide–semiconductor) image sensor. Therefore, our system was computed by four orders of Zernike coefficients with 10 aberration terms and each centroid position was calculated by the Labview^®^ program. The comparison between the reference focal spots and the aberrated focal spots was shown in Figure 9(c), two previous pictures stacking, where red rectangles are the detected reference spots position and green rectangles were the detected aberrated spots position. The displacement was then calculated with the equations listed above.

**Figure 10.**
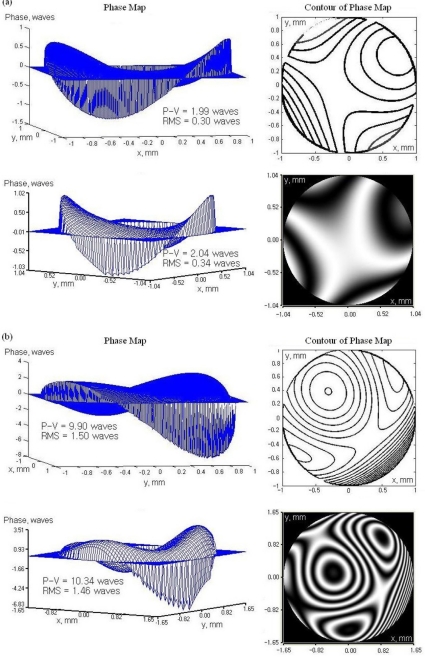
Wavefront measurement results of **(a)** PAL and **(b)** PCX by our SHWS (above) and commercial SHWS (below).

The wavefront aberration generated by the testing lens was measured by both our SHWS and a commercial wavefront sensor from OKO Tech (UI2210-m, UEye, NL). The lenslet array in the commercial wavefront sensor is in hexagonal configuration and the pitch size is 300 μm. To evaluate the performance of double layer microlens arrays, we compared the measurement result between our SHWS and the commercial one. First, we examined the PAL which is used to compensate the presbyopia. SHWS is used to observe the change of planar wavefront caused by lenses. Second, we observed the aberration caused by titled incidence wavefront through PCX where rays pass through the lens at an angle to the axis θ. Figure 10 shows the measurement results by a three-dimensional phase map and its contour.

Because the reference and the distorted beams pass through exactly the same optical path in this system, the aberrations are eliminated by the reference beam. Thus, the wavefront of the optical objectives can be measured precisely. The displacements of the reference spots and the aberrated spots are detected by a CMOS image sensor and processed by the Labview^®^ program on a personal computer. The calculation of Zernike coefficients and wavefront reconstruction are performed by Matlab^®^.

The wavefront measurement accuracy of our SHWS was evaluated by a comparative measurement with the commercial wavefront sensor. Unlike our SHWS that calculated 37 wavefront slopes, the commercial SHWS automatically chose the brightest centroid spots to calculate aberrations. The number of spots found is variable; it depends on two parameters- the intensity threshold (with respect to the intensity of the brightest spot) and the upper limit of maximum number of spots. The commercial program searches for spots in the reference pattern and builds bounding boxes around them. Then, spots of the main pattern are searched within the bounding boxes calculated from the reference pattern. Our SHWS apply the same technique; however, the calculated number of spots is fixed to 37.

As shown in Figure 10(a), from a review of the coefficient and the contour map, we noticed that the astigmatism aberration dominates in which light in one plane (the “plane of the paper” or meridional plane) focused at different location from the orthogonal plane. The root-mean-square (RMS) value of the wavefront aberration calculated by the commercial SHWS is 0.336λ (λ = 630 nm) while the RMS value calculated by our SHWS is 0.3λ. The measurement difference is less than λ/25 in RMS. Figure 10(b) shows the result for testing a PCX lens. The above plot shows the measured wavefront which resulted in combination of coma and astigmatism aberration, nevertheless the coma aberration still dominates as expected and the plot below shows the expected wavefront taken by the commercial SHWS, and. The peak to valley difference is 0.438λ and the RMS wavefront difference is 0.036λ. The commercial SHWS calculated 19 wavefront slopes. Table 3 shows the comparison of wavefront measurement results between commercial SHWS and our SHWS.

We believe that high sensitive wavefront sensors could be used in measuring dynamic deformation of microstructures under high operation frequency [10]. There, however, is a tradeoff between dynamic range and sensitivity according to the number of microlenses and the focal length [11]. A small-size, long-focal-length MLA could increase the measurement accuracy associated with Equation (5) [8]. A longer focal length will provide high sensitivity in determining the average slope across each lenslet under a given wavefront [12]. Nevertheless, a microlens array with a shorter focal length will have greater dynamic range at the cost of reduced sensitivity. According to Equation (1), the focal length of microlens array is related to the radius of curvature of the lens. In order to produce a short or long focal length MLA, the size of the lenslet need to be redesigned. However, by our new proposed method, we do not have to redesign and fabricate new microlenses; different focal lengths can be realized by simply modifying the refractive index difference without changing the MLA size. The master mold could be reused to replicate new optical transparent polymers and different MLA materials could be applied, which results in different focal length MLAs. Hence, we could build distinctive characteristic SHWS according to different applications.

**Figure 11.**
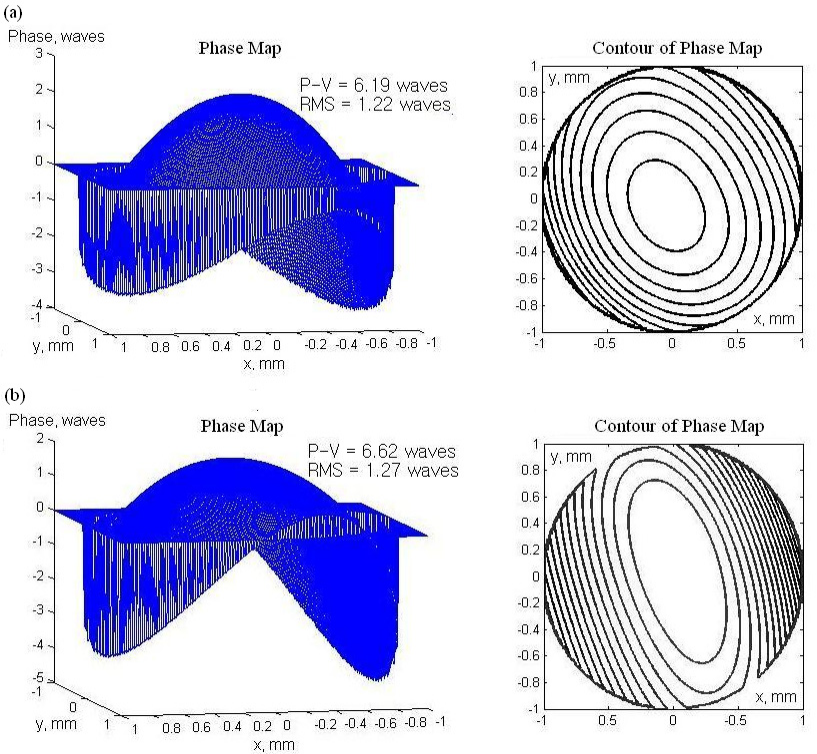
Wavefront measurement results of defocus by using **(a)** long focal length (UV-resin) and **(b)** shorter focal length (SU-8) MLA as our SHWS lenslet component.

In order to verify the feasibility of this technique, we also fabricated an SU-8 (n = 1.59) polymer MLA which has shorter focal length of 2.6 mm. Figure 11 shows the wavefront measurement compared between the previous built SHWS with long focal length and the new SHWS with SU-8 MLA as the lenslet component. Both evaluated wavefronts were dominated by defocus aberration which corresponds to the parabola-shaped optical path difference between two wavefronts that was tangent at the vertices and had different curvature radii. The peak-to-valley difference between long focal length MLA SHWS and shorter focal length MLA SHWS is 0.433λ. The RMS wavefront of the long focal length MLA SHWS is λ/20 less than the shorter focal length MLA. In other word, long focal length MLA SHWS has higher accuracy and sensitivity than the expected 1.17λ wavefront in rms. As a result, another advantage of the proposed microlens is that we can make different MLAs without changing lens curvature or pitch size. That means that the spatial resolution can be kept the same while the sensitivity is changed.

## 5. Conclusions

A simple and easy method to extend the focal length of MLAs was experimentally demonstrated. A covered PDMS layer was room-temperature cured upon the MLA. Because of the reduced refractive index difference, the light is less refracted and this results in a long focal length. We used the UV-resin microlens to implement the MLA and other materials, such as SU-8, can be employed with the same concept, resulting in a shorter focal length MLA. Different focal lengths could be available using the same master mold by simply modifying the refractive index of the microlens material. In other words, we can achieve different MLAs without changing the curvature or pitch distance. The method in this paper also provides a significant increment in the focal length and the development of long focal length MLAs has been successful both using UV-resin and SU-8 as the MLA material.

The fabricated MLAs were also compatible with a CMOS image sensor in constructing wavefront sensors. The wavefront measurement accuracy of our wavefront sensor was evaluated by a comparative measurement using a commercial wavefront sensor, which was less than λ/25 in RMS difference. On the other hand, we also compared a long focal length MLA and a shorter focal length MLA produced by the same method with different refractive index difference. The wavefront measurement result verifies that the long focal length MLA has higher accuracy and sensitivity and its RMS is λ/20 better than that of the shorter focal length MLA. Hence, our MLA fabrication method has high reproducibility and could be implemented to build SHWS with different specifications. Spatial resolution remains the same while sensitivity is changed. The proposed double layer microlens was proven to be suitable for wavefront sensing applications.

## Figures and Tables

**Table 1.** Lens arrangement versus fill factor for 20 μm of lens gap.

**Lens Arrangement**	**Lens Area (mm^2^)**	**Total Area (mm^2^)**	**Fill Factor**
Circular lens quadratic grid (d = 135 μm)	0.0143	0.0240	59.58%
Circular lens hexagonal grid (d = 235 μm)	0.0434	0.0563	77.09%
Hexagonal lens (d = 235 μm)	0.0478	0.0563	84.9%
Square lens (d = 235 μm)	0.0552	0.0650	84.9%

**Table 2.** Methods for increasing the focal length of MLA.

**Methods**	**Advantage**	**Disadvantage**
Refractive index difference	Significant increment (6X longer)	Double layer (2^nd^ PDMS coating is easy)
Increase refractive index, n	Single layer process	Slightly (∼1X, Depends on material property)
Increase diameter	Single layer process	Results in thick photoresist and spin coating problems

**Table 3.** The comparison of wavefront measurement by our SHWS and commercial SHWS.

**Lens**	**RMS (λ = 630 nm)**		**Peak-to-Valley (λ = 630 nm)**		**Wavefront Aberration**

	**our SHWS**	**commercial SHWS**	**our SHWS**	**commercial SHWS**	
PAL	0.3λ	0.336λ	1.987λ	2.043λ	Astigmatism
PCX	1.5λ	1.464λ	9.903λ	10.341λ	Coma
